# Image-Driven Hybrid Structural Analysis Based on Continuum Point Cloud Method with Boundary Capturing Technique

**DOI:** 10.3390/s25020410

**Published:** 2025-01-11

**Authors:** Kyung-Wan Seo, Junwon Park, Sang I. Park, Jeong-Hoon Song, Young-Cheol Yoon

**Affiliations:** 1 Department of Civil Engineering, Myongji College, Seoul 03656, Republic of Korea; k.w.seo@yonsei.ac.kr (K.-W.S.); william@willreal.tech (J.P.); 2 Research Institute for Safety Performance, Korea Authority of Land & Infrastructure Safety, Jinju 52856, Republic of Korea; s.park@kalis.or.kr; 3 Department of Civil Environmental and Architectural Engineering, University of Colorado, Boulder, CO 80309, USA; jh.song@colorado.edu

**Keywords:** hybrid structural analysis, continuum point cloud method, digital image processing, polynomial regression, essential boundary condition, boundary value problem

## Abstract

Conventional approaches for the structural health monitoring of infrastructures often rely on physical sensors or targets attached to structural members, which require considerable preparation, maintenance, and operational effort, including continuous on-site adjustments. This paper presents an image-driven hybrid structural analysis technique that combines digital image processing (DIP) and regression analysis with a continuum point cloud method (CPCM) built on a particle-based strong formulation. Polynomial regressions capture the boundary shape change due to the structural loading and precisely identify the edge and corner coordinates of the deformed structure. The captured edge profiles are transformed into essential boundary conditions. This allows the construction of a strongly formulated boundary value problem (BVP), classified as the Dirichlet problem. Capturing boundary conditions from the digital image is novel, although a similar approach was applied to the point cloud data. It was shown that the CPCM is more efficient in this hybrid simulation framework than the weak-form-based numerical schemes. Unlike the finite element method (FEM), it can avoid aligning boundary nodes with regression points. A three-point bending test of a rubber beam was simulated to validate the developed technique. The simulation results were benchmarked against numerical results by ANSYS and various relevant numerical schemes. The technique can effectively solve the Dirichlet-type BVP, yielding accurate deformation, stress, and strain values across the entire problem domain when employing a linear strain model and increasing the number of CPCM nodes. In addition, comparative analysis with conventional displacement tracking techniques verifies the developed technique’s robustness. The proposed technique effectively circumvents the inherent limitations of traditional monitoring methods resulting from the reliance on physical gauges or target markers so that a robust and non-contact solution for remote structural health monitoring in real-scale infrastructures can be provided, even in unfavorable experimental environments.

## 1. Introduction

Structural health monitoring (SHM) and deformation analysis play a vital role in maintaining the safety and functionality of infrastructure. Due to various loads, environmental influences, and aging, modern engineering structures demand reliable methods to effectively evaluate deformation, stress, and strain for appropriate operation and maintenance [1,2,3]. Conventionally, these kinematic variables are measured using contact sensors or non-contact devices tracking predefined targets [4,5,6]. Contact sensors, such as strain gauges and fiber optic sensors, are known for their high precision and real-time feedback capabilities. However, they often meet challenges, such as the extensive resources required for data processing and issues with hostile sensor installation environments on actual infrastructures [7]. On the other hand, non-contact sensing approaches suggest interesting resolutive features, such as structural methodology couplings or nonphysical target implementation, offering viable alternatives for kinematic variable measurement [8,9,10,11].

In many practical cases, special treatment is required to extrapolate measured or computed kinematic values across an entire structure, which demands substantial computational exertion and time [12,13,14]. To avoid tricky situations and enrich the performance of traditional sensor utilization, considerable research has favored methods that merge FEM with non-contact sensing tools [15,16,17,18]. This combination tries to emulate the kinematic variables from displacement data gathered from direct and indirect sources [19,20,21,22,23,24,25]. Typically, during the combination, an FEM mesh is synchronized with the data measurement configuration, and the synchronization mainly focuses on numerically computed displacements, so kinematic variable calculation using FEM requires frequent adjustments to the mesh, especially when monitoring intricate deformations such as large deformation and crack-induced displacement. In this scenario, non-mesh techniques like mesh-free methods become essential, especially when the mechanical calculations need to be conducted at an arbitrary position outside predefined nodes or mesh grids. Mesh-free techniques, such as the boundary element method (BEM) [26,27] or the element-free Galerkin method (EFGM) [28,29], have emerged as alternatives to FEM to avoid mesh-connectivity-induced difficulties. In addition, recent studies have explored graph-based models, such as graph neural networks (GNN) [30] and vision-based techniques using single-camera systems [31], to manipulate high-fidelity numerical simulation combined with a data-driven machine learning approach and introduce a deep neural network designed to predict 3D mesh deformation directly from a single image input, combined with an initial 3D cube mesh input, respectively. However, these methods might rely on data-driven models or require complex calibration. However, the BEM is unsuitable for dealing with significant deformation inside the boundary element model, and the fundamental solution does not seem quite feasible for handling experimental data. Although the EFGM is included in the category of representative mesh-free techniques, it still requires the integration cell to integrate the weak form, making itself partially dependent on mesh structures [28,29]. In addition, its derivative approximation computation demands quite a complicated process and considerable computational resources. For this reason, a strong-form-based mesh-free method has been strongly recommended as a remedy [32,33,34]. It has been proven to have proper flexibility to provide digital image processing (DIP) for structural analysis based on a truly mesh-free platform, as seen in [35].

Park et al. [35] proposed a synthetic structural analysis scheme that combines the DIP with a strong-form particle method called PDM. The scheme employed displacement image data from targets attached to the real structure surface to simulate a real-scale experiment without solving governing partial differential equations. Although it manipulated PDM’s node-wise displacement data to enhance the accuracy of kinematic variable tracking, it inevitably seriously relies on the target configuration and raw data quality. On the other hand, Seo et al. [36] presented an edge shape tracking technique that utilizes light detection and ranging (LiDAR) without preset targets on the specimen during the loading process. The technique recognizes the edge shape change due to the loading and transforms the change into essential boundary conditions for the numerical analysis based on the PDM. Unlike Park et al. [35], the boundary value problem (BVP) formulated as a partial differential equation set should be solved to obtain the mechanical variables. Seo et al. [36] noted that the captured boundary deformations can be transformed into the essential boundary conditions for the Dirichlet-type BVP.

This study proposes a technique that captures the essential boundary condition using a digital image photographed from the real flexural loading test for a rubber beam and polynomial regression analysis. The BVP is equipped with the essential boundary condition and is solved by the strong-form-based mesh-free method called CPCM. Kinematic variables such as displacement, strain, and stress are obtained from the BVP solutions. Unlike Park et al. [35] and Seo et al. [36], the developed technique directly solves the BVP without involving target displacement data and utilizes the digital image for polynomial regression, respectively. The feasibility and accuracy of resultant kinematic variables are verified by comparing them with reference data and the results obtained by other relevant methods inspired by the methodologies of Park et al. [35] and Seo et al. [36].

## 2. Strongly Formulated Boundary Value Problem

### 2.1. Derivative Approximation Derivation

Derivative approximations are essential in solving the strongly formulated BVP. As referred to in [37], the PDM uses only nodes or particles to solve the BVP. The CPCM shares the same mathematical background as the PDM. It deals with a continuum body based on the neighbor cloud particles [37]. The governing equations are solved at every particle according to the particle’s region, for example, the equilibrium equation for the interior nodes and the essential boundary equation for the boundary nodes. More specifically, based on the moving least squares method, the Taylor series is numerically expanded at the particles using neighbor cloud particles encompassed in the influence domain. In this process, shape functions and their derivatives are derived without any differentiation calculation. The order of the polynomial basis vector and the number of neighbor cloud particles affect the accuracy of the derivative approximation. Unlike the grid in the finite difference method, the mesh in FEM, or the integration cell in other meshless methods, the CPCM does not suffer from any restrictions of the structured grid or mesh or cell; additionally, its particle arrangement or even the number of particles can change during the analysis. Thus, the CPCM can sophisticatedly avoid edge extraction difficulties due to the persistence of the numerical model, such as the finite element mesh or the finite difference method grid.

In strongly formulated solid mechanics problems, the governing equations are often discretized in terms of displacement, leading to Navier’s equation, which necessitates the second-order derivatives of displacements or the divergence of stress [34]. On the other hand, this study employs an improved numerical scheme that facilitates only first-order derivatives, as in Yoon et al. [37]. The stress divergence is discretized by consecutively using the first-order derivatives, achieving improved precision and efficiency. The following part presents a concise overview of the derivative approximation construction as detailed in [37].

A Taylor polynomial at an arbitrary position ***x*** against the reference point ***y*** based on the *m*th polynomial vector can be written as follows:

(1) $$u_{i}(x;y)=p_{m}^{T}(x,y)a_{i}(y)=\left(\frac{{\left(x-y\right)}^{\alpha_{1}}}{\alpha_{1}!},\;\cdots ,\;\frac{{\left(x-y\right)}^{\alpha_{K}}}{\alpha_{K}!}\right)\cdot \left(\begin{array}{c} D_{x}^{\alpha_{1}}u_{i}\left(y\right)\\ \;\;\vdots \;\;\\ D_{x}^{\alpha_{K}}u_{i}\left(y\right) \end{array}\right)$$

where $D_{x}^{\alpha }=\partial_{x_{1}}^{\alpha_{1}}\cdots \partial_{x_{n}}^{\alpha_{n}}$ represents the differential operator expressed in terms of multi-index natation; then, $\alpha_{1}=\left(0,\cdots ,0\right)\;$ and $\alpha_{K}=\left(0,\cdots ,m\right)$. $p_{m}^{T}(x,y)\;$ represents the *m*th order polynomial basis vector, and $a_{i}(y)$ is the derivative coefficient vector for spatial component i=(0,⋯,n). *n* represents the space dimension, and $K=\frac{\left(m+n\right)!}{m!n!}$. When there are *N* particles included in the influence domain of the reference point ***y***, Equation (1) is related to the nodal solution $u_{iI}=u_{i}\left(x_{I}\right)$ by using the residual of the moving least squares method as follows:

(2) $$J=\sum_{I=1}^{N}\omega_{I}(y){\left(p_{m}^{T}(x_{I},y)a_{i}(y)-u_{iI}\right)}^{2}$$

where $\omega_{I}(y)=\omega \left(\frac{\left|x_{I}-y\right|}{r}\right)$ is the weight function, and *r* denotes the radius of influence domain that determines how many neighbor particles are included in the derivative approximation calculation. Minimizing Equation (2) is achieved by considering a stationary condition ($\frac{\partial J}{\partial a}=0$), yielding $a_{i}(x)$, as follows:

(3) $$a_{i}(x)={\left(\sum_{I=1}^{N}\omega_{I}(x)p_{m}(x_{I},x)p_{m}^{T}(x_{I},x)\right)}^{-1}\cdot \left(\omega_{1}(x)p_{m}(x_{1},x),\cdots ,\omega_{N}(x)p_{m}(x_{N},x)\right)\cdot {u_{i}}^{h}$$

where $u_{i}^{h}={\left(u_{i1},\cdots ,u_{iN}\right)}^{T}$ is a vector that collects nodal solutions, and the ***y*** of Equation (2) can be replaced by ***x*** via the moving process [15,16]. Recall that the $a_{i}\left(x\right)$ contains all the derivative approximations from the zeroth order to *m*th order, corresponding to the Taylor polynomial in Equation (1). By rearranging Equation (3) in a matrix form, we have the following expression for the shape function, its derivatives, and the nodal solution:

(4) $$a_{i}(x)=\left(\begin{array}{ccc} \phi_{1}^{\left[\alpha_{1}\right]}\left(x\right) & \cdots & \phi_{N}^{\left[\alpha_{1}\right]}\left(x\right)\\ \vdots & \ddots & \vdots \\ \phi_{1}^{\left[\alpha_{K}\right]}\left(x\right) & \cdots & \phi_{N}^{\left[\alpha_{K}\right]}\left(x\right) \end{array}\right)\cdot \left(\begin{array}{c} u_{i1}\\ \vdots \\ u_{iN} \end{array}\right)$$

Recall from Equation (1) that $a_{i}(x)=\left(D_{x}^{\alpha_{1}}u(x),\cdots ,D_{x}^{\alpha_{K}}u(x)\right)$. In the above, $\phi_{I}^{\left[\alpha_{1}=(0,0)\right]}(x)$ and $\phi_{I}^{\left[\alpha_{K}=(0,m)\right]}(x)$ denotes the zeroth and $\alpha_{K}$th order shape functions for particle *I* at ***x*** for the two-dimensional case, respectively. In the above, since the first row is the collection of the zeroth order shape functions, the *i* directional displacement value at ***x*** point is then calculated by $u_{i}^{h}\left(x\right)=\sum_{I=1}^{N}\phi_{I}^{\left[\left(0,0\right)\right]}(x)u_{iI}$. It can be stressed that calculation of $a_{i}\left(x\right)$ requires no actual differentiation; all derivative approximations up to the *m*th order are simultaneously computed by matrix inversion in Equation (3). More detailed derivation procedures for these derivative approximations can be found in [34,37].

### 2.2. Governing Equation Discretization and Kinematic Variable Calculation

**Figure 1:**
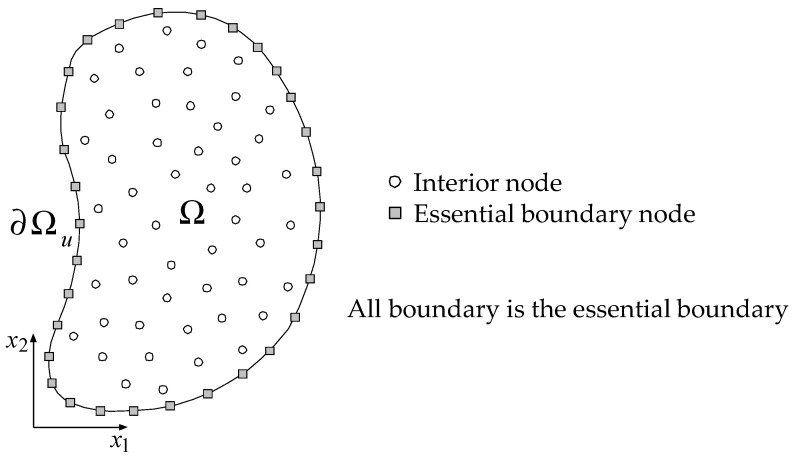
Dirichlet-type BVP configuration modeled with interior and boundary nodes.

During the structural loading test, deformation data of the specimen edge are gathered from constant-time-interval photoshoots using a high-resolution digital camera. Subsequently, DIP and regression analysis are carried out to quantify the essential boundary value due to deformation. As a result, all boundary values can be prescribed, and the Dirichlet-type BVP can be defined entirely for the given problem domain. Commonly, the BVP comprises both essential and natural boundary conditions in solid mechanics problems, but the BVP is set up only using an essential boundary condition in this study. Figure 1 shows the computational domain discretized with interior and boundary nodes. The equilibrium equation is solved at each interior node, and the captured essential boundary conditions are assigned to the boundary nodes. After solving the BVP using the CPCM, kinematic variables such as displacement and strain are computed for all interior nodes using Equation (4). In addition, the stress can be easily calculated in the same manner as in FEM using a constitutive equation.

A brief explanation of the CPCM formulation for a solid body is given here; more details can be found in Yoon et al. [38]. When the deformation occuring in the body is small, and the problem is static, the equilibrium equation to be solved for the interior nodes is written as

(5) $$\sigma_{ij,j}+b_{i}=0\;\mathrm{in}\Omega$$

where $\sigma_{ij}$ represents the Cauchy stress tensor, and $b_{i}$ stands for the body force. Since the stress tensor includes first-order derivatives of displacement, the equilibrium equation finally involves the second-order derivatives. Since elastic material is considered in this study, the constitutive equation is given as

(6) $$\sigma_{ij}=\lambda \varepsilon_{kk}\delta_{ij}+2\mu \varepsilon_{ij}$$

where μ and λ are Lamé constants, $\varepsilon_{ij}$ is the strain tensor, and $\delta_{ij}$ is a second-order identity tensor. In the small deformation problem, the strain is expressed as the symmetric part of the displacement gradient as follows:

(7) $$\varepsilon_{ij}=\frac{1}{2}\left(u_{i,j}+u_{j,i}\right)$$

When the body force is absent, the equilibrium equation of Equation (5) can be expressed in a matrix form as below:

(8) $${B\left(x\right)}^{T}DB\left(x\right)u\left(x\right)=0$$

where $u\left(x\right)={\left(u_{11}\left(x\right),\cdots ,u_{2N}\left(x\right)\right)}^{T}$. The above equation needs to be assembled over all the interior nodes during the construction of the stiffness matrix of the total system. The strain and stress are calculated based on Equations (6) and (7) using the computed displacements and derivative approximations, respectively; i.e., $\varepsilon_{ij}=B\left(x\right)u\left(x\right)$ and $\sigma_{ij}=DB\left(x\right)u\left(x\right)$, where the $B\left(x\right)$ matrix is constructed using the first-order derivatives of the shape function, and D matrix describes the material properties given in Equation (6), respectively, as follows:

(9) $$B(x)=\sum_{I=1}^{N}\left[\begin{array}{cc} \varphi_{I}^{\left[\left(1,0\right)\right]}(x) & 0\\ 0 & \varphi_{I}^{\left[\left(0,1\right)\right]}(x)\\ \varphi_{I}^{\left[\left(0,1\right)\right]}(x) & \varphi_{I}^{\left[\left(1,0\right)\right]}(x) \end{array}\right]$$

(10) $$D=\left[\begin{array}{ccc} 2\mu +\lambda & \lambda & 0\\ \lambda & 2\mu +\lambda & 0\\ 0 & 0 & \mu \end{array}\right]$$

The essential boundary condition for the Dirichlet-type BVP is given by

(11) $$u_{i}=\bar{u_{i}}\mathrm{on}\;\partial \Omega_{u}$$

where $\bar{u_{i}}$ denotes the prescribed boundary value. Note that the value of the structure’s edge deformation obtained from the DIP and regression analysis is assigned using Equation (12) as the essential boundary condition. Namely, Equation (11) is implemented for all the boundary nodes by the following matrix form:

(12) $$I\left(x\right)u\left(x\right)=\bar{u_{i}}$$

where $I\left(x\right)$ is an interpolation operator that includes the zeroth order shape function in its diagonal slots and is discretized with *N* neighbor particles as follows:

(13) $$I(x)u(x)=\sum_{I=1}^{N}\left[\begin{array}{cc} \varphi_{I}^{\left[\left(0,0\right)\right]}\left(x\right) & 0\\ 0 & \varphi_{I}^{\left[\left(0,0\right)\right]}\left(x\right) \end{array}\right]\left(\begin{array}{c} u_{1I}\\ u_{2I} \end{array}\right)$$

The CPCM assembles all discrete governing equations given in Equations (8) and (12) for all the nodes. The assemblage for all nodes yields a final system of equations for Dirichlet-type BVP. When dealing with the essential boundary condition, unlike other weak-form-based mesh-free methods, any enforcement using the constraint formulation, such as the Lagrange multiplier method and penalty method, is not necessary [28]. In addition, unlike the FEM, it is not required to send the known boundary values to the right-hand side of the system together with the corresponding stiffness components. The total system of equations is written in an algebraic equation form as follows:

(14) $$\left[\begin{array}{c} A_{I=1}^{N}\left(B_{I}^{T}D_{I}B_{J}\right)\\ A_{K=1}^{M}\left(I_{K}\right) \end{array}\right]\left[\begin{array}{c} u_{11}\\ \vdots \\ u_{2N} \end{array}\right]=\left[\begin{array}{c} 0\\ A_{K=1}^{M}\left({\bar{u}}_{K}\right) \end{array}\right]$$

where $A_{I=1}^{N}$ refers to an assemblage of the equilibrium equations for interior nodes 1 to *N*, and $A_{K=1}^{M}$ represents an assemblage of the essential boundary equations for boundary nodes 1 to *M*. The stiffness matrix is constructed by assembling discrete equations according to the original node number. The assembly does not need to be conducted separately for the internal and boundary nodes. Therefore, the discrete governing equations can be easily assembled according to the original node number.

## 3. Experimental and Simulation Verifications

### 3.1. Experimental Configuration and Image-Driven Edge Shape Capturing

**Figure 2:**
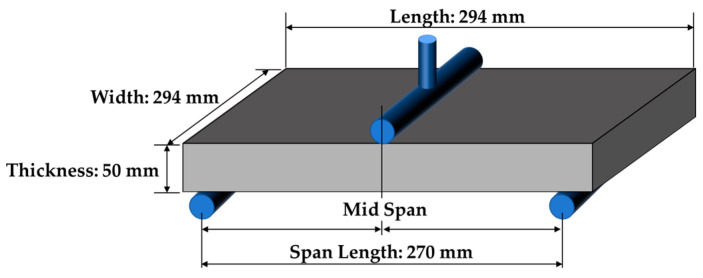
Configuration of three-point bending test for rubber beam.

**Figure 3:**
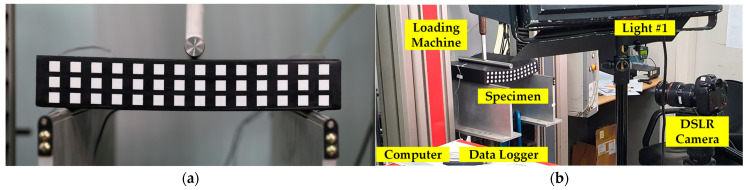
Experimental setup for digital image acquisition of the 3-point bending test: (**a**) specimen setup; (**b**) device setup.

Digital images used for experimental verification were obtained from the experiment conducted by Park et al. [35]. In the experiment, a simply supported rubber beam under a three-point bending condition was loaded and digitally photographed (see Figure 2 and Figure 3). In the simulation, Young’s modulus of *E* = 2 MPa and Poisson’s ratio of *v* = 0.45 were assumed. For the digital image acquisition, a Canon 5D Mark IV (Tokyo, Japan) full-frame camera with a resolution of 30.1 million pixels (6720 pixels wide × 4480 pixels long) equipped with a Canon EF 24-70 F2.8L II lens (Tokyo, Japan) was used. Snapshots of the experimental setup are given in Figure 3a,b. In this setting, the unit pixel size was approximately 0.063 mm. The external load increased steadily at 0.11 mm/s, reaching 2046.5 *N*, while the maximum deflection was limited to 50 mm. Images were taken and stored every 2 s to capture the specimen’s deformation. Although the images, including the ordered square targets, were acquired in this study, the target images are not used for edge tracking; the specimen’s edge images are selectively used. Note that the BVP analysis of this study employs the digital image of the deformed beam only to capture the edge shape change. The image taken when the central deflection was 2.64 mm is used for simulation since the rubber beam is regarded to be in an elastic stage up to that point. In addition, a similar experiment can be found in Seo et al. [36].

**Figure 4:**
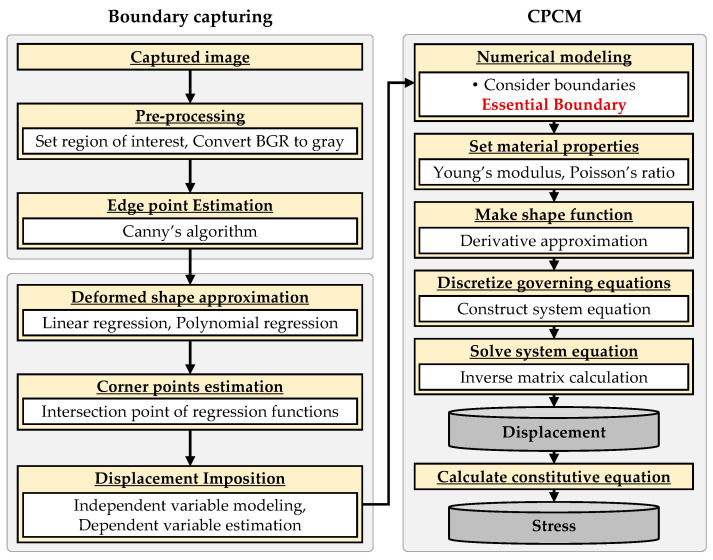
The procedure of estimation and assignment of the essential boundary value from the digital image to the CPCM simulation.

**Figure 5:**
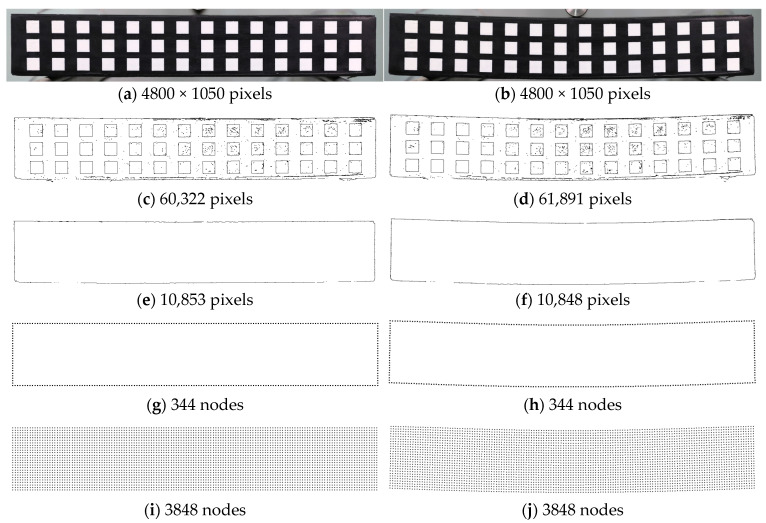
DIP-based boundary capturing results by the steps outlined in Figure 4: (**a**) the initial state image (step 1); (**b**) the deformed shape image (step 1); (**c**) 2D edge pixels (step 2 for the initial state); (**d**) 2D edge pixels (step 2 for the deformed specimen); (**e**) 2D edge pixels (step 3 for the initial state); (**f**) 2D edge pixels (step 3 for the deformed specimen); (**g**) captured essential boundary (step 3 for the initial state boundary nodes); (**h**) captured essential boundary (step 3 for boundary nodes after loading simulation); (**i**) total CPCM model for initial state; (**j**) total CPCM model after loading simulation.

Figure 4 illustrates the boundary capturing procedure for estimating the edge points of the deformed specimen from the digital image and applying essential boundary values to the CPCM analysis. The digital image is handled in jpg file format, and the entire process was carried out in a Python 3.11 environment using the OpenCV 4.5.5 [39], Open3D 0.16.0 [40], and SciPy 1.7.3 [41] libraries. As shown in Figure 5a,b, Step 1 involves ROI (region of interest) determination processing from the initial digital image for the specimen. Step 2 explains how to determine the edge points of the deformed specimen image. First, the Canny edge detection algorithm [42] was applied. The results are shown in Figure 5c,d. The edge points were successfully captured to be implemented in the CPCM model as essential boundary conditions. However, it is important to note that Canny’s edge detection algorithm may be sensitive to noise and lighting variations, potentially affecting its robustness in real-world applications. This process estimates edges by detecting pixel value change, i.e., after removing other outliers, edge points are specified regardless of the attached targets on the specimen surface. As a result, edge points for the boundary of the deformed specimen were extracted, as shown in Figure 5e,f. Step 3 involves applying essential boundary values to the CPCM boundary nodes (See Figure 5g,h). Figure 5i,j represents the CPCM analysis models before and after loading simulation. When comparing these with the results obtained using the LiDAR point cloud data [1], it is confirmed that the Canny edge estimation algorithm extracts more qualified edge points (pixels) from the digital image. Regression analysis can reduce the overfitting risk by interpolating the deformed edge shape. In addition, note that fewer errors occur as the degree of the polynomial regression equation increases.

### 3.2. Essential Boundary Capturing Using Polynomial Regression

The extracted edge points need to be transformed into the essential boundary values for boundary nodes of the CPCM model. A one-dimensional polynomial regression equation is applied to track the shape changes of the deformed boundary as follows:

(15) $$y=\beta_{0}+\beta_{1}x+\beta_{2}x^{2}+\ldots +\beta_{n}x^{n}=\beta^{T}\cdot p\left(x\right)$$

where *x* is the independent variable, meaning the relative coordinate along an edge axis; *n* is the highest degree of the polynomial regression equation; $\beta^{T}={(\beta }_{0},\beta_{1},\ldots ,\beta_{n})$ is the coefficient vector of the regression equation; and $p^{T}\left(x\right)=\left(1,x,\ldots ,x^{n}\right)$ is the one-dimensional polynomial vector to describe the shape of the deformed boundary.

**Figure 6:**
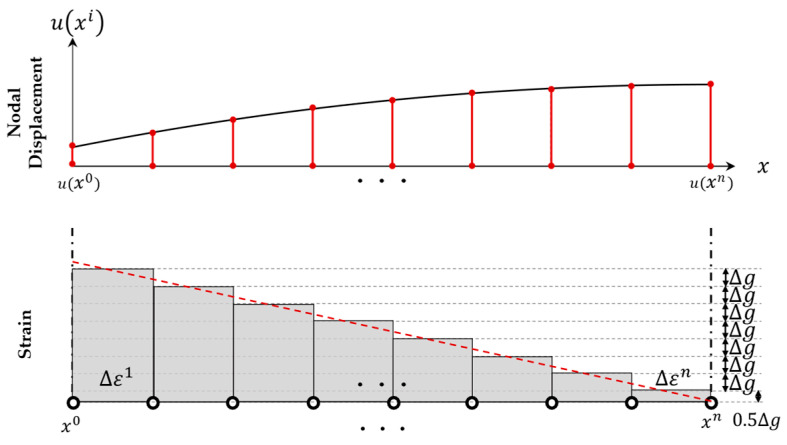
Edge displacement interpolation along the boundary nodes based on linear strain model with zero strain at $x^{n}$ and end deformations $u(x^{0})$ and $u\left(x^{n}\right)$.

Seo et al. [36] proposed a simple interpolation method for determining the displacement of boundary nodes of the CPCM model in two-dimensional BVP. The method employs a linear strain model to estimate the coordinates of edge-axis nodes of the deformed specimen. When the edge-axis is coaxial with the *x*-axis, the independent variable coordinates $x_{ind}^{i}$ of the regression equation can be expressed as follows:

(16) $$x_{ind}^{i}=x+u\left(x^{i}\right)$$

where the superscript denotes the number of the edge nodes, starting from zero and ending at *n*, and $x_{ind}^{i}$ represents the coordinates after deformation at the *i*th node along the edge-axis. Figure 6 illustrates the method of calculating the edge-axis displacement when considering the linear strain at an arbitrary position for the end values, $u\left(x^{0}\right)$ and $u\left(x^{n}\right)$, with zero strain at $x^{n}$. For more straightforward computation, the strain is assumed to be constant within a section divided into equal size, and the value at the center of the section is taken.

The edge-axis displacement can then be expressed as a quadratic function of $x^{i}$, as follows:

(17) $$u\left(x^{i}\right)=a{\left(x^{i}\right)}^{2}+bx^{i}+c,(x^{0}\leq x^{i}\leq x^{n})$$

where *a*, *b*, and *c* are coefficients of the quadratic polynomial $u\left(x^{i}\right)$, and in fact, c denotes the displacement at the starting point $u\left(x^{0}\right)$. For example, if the edge-axis is coaxial with the *x*-axis and the spacing of boundary nodes of the CPCM model is uniform, the nodal displacement $u\left(x^{k}\right)$ of the *k*th node can be calculated as the cumulative sum of strains by Equation (18). More details can be found in Seo et al. [36]

(18) $$u\left(x^{k}\right)=u\left(x^{0}\right)+\int_{x^{0}}^{x^{k}}\frac{du\left(x^{i}\right)}{dx}dx\approx u\left(x^{0}\right)+\Delta \varepsilon^{1}+\ldots +\Delta \varepsilon^{k}=u\left(x^{0}\right)+\sum_{j=1}^{k}\Delta \varepsilon^{j}$$

### 3.3. BVP Analysis Results Using the CPCM Equipped with Boundary Capturing Technique

**Figure 7:**
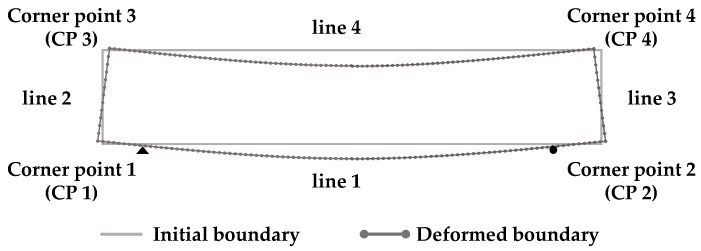
Essential boundary configuration for the rubber beam specimen (edge and corner point numbering).

This section provides image-driven hybrid structural analysis results using the CPCM with the boundary capturing technique. The results are also compared with those obtained by the conventional methods, including the target-based DIP method, and the accuracy of the displacement boundary capture based on the polynomial regression equation is demonstrated together. The first analysis was performed using a complete numerical simulation using commercial structural analysis software, ANSYS 2020, under the same conditions as the three-point bending experiment. This full ANSYS simulation result is for comparison and a posteriori error analysis. The second analysis was conducted by applying the essential boundary condition captured by the regression equation using the edge points extracted from the ANSYS analysis result regarding the deformed boundary shape, where ANSYS only provides the shape of boundary deformation. The CPCM performed the third analysis, equipped with the boundary capturing technique consisting of DIP and polynomial regression analysis. The edges of the beam specimen were numbered in the same manner as in Seo et al. [36]. As seen in Figure 7, lines 1 and 4 are approximated by the second- to fifth-degree polynomial regression equations; the linear and uniform strain models are selected for the edge displacement capturing.

**Figure 8:**
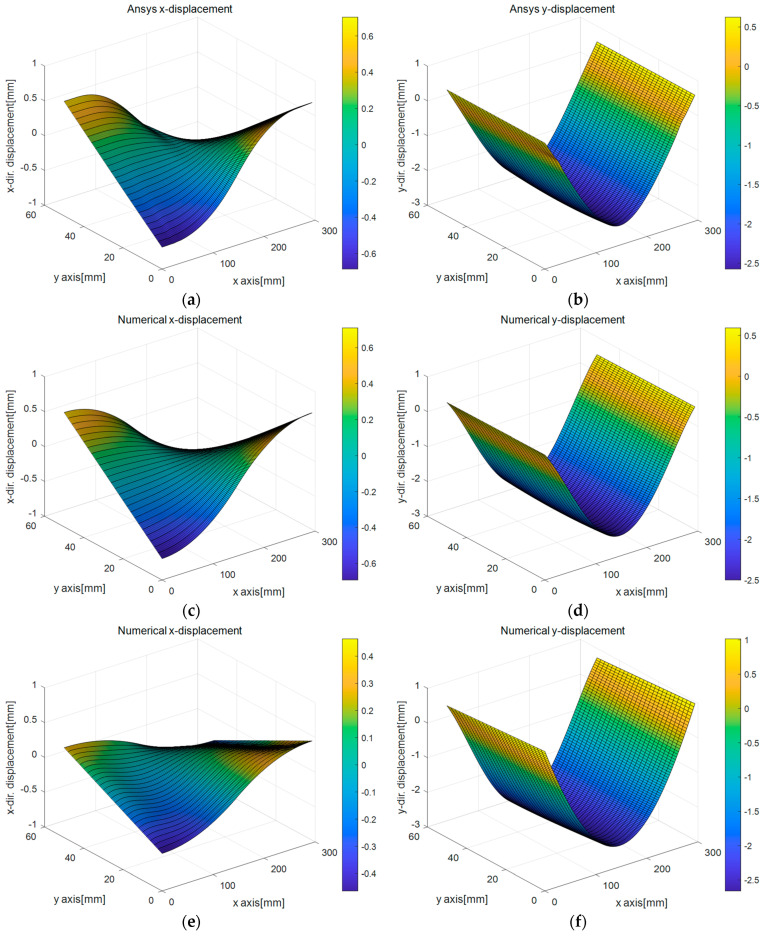

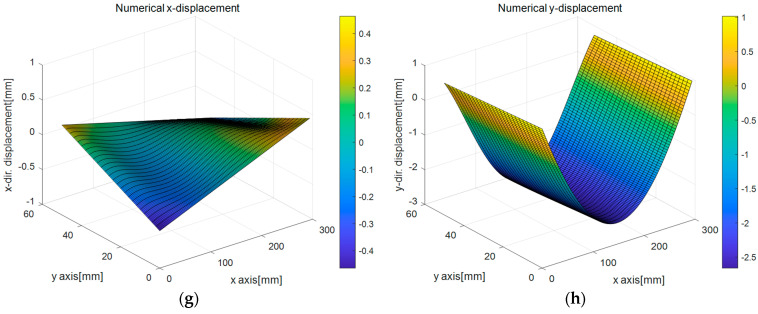
Surface plots for computed displacements obtained by different simulation methods and boundary capturing schemes for 3-point rubber beam bending test: (**a**) $u_{x}$ (Method 1), (**b**) $u_{y}$ (Method 1), (**c**) $u_{x}$ (Method 2), (**d**) $u_{y}$ (Method 2), (**e**) $u_{x}$ (Method 3), (**f**) $u_{y}$ (Method 3), (**g**) $u_{x}$ (Method 4), (**h**) $u_{y}$ (Method 4).

**Figure 9:**
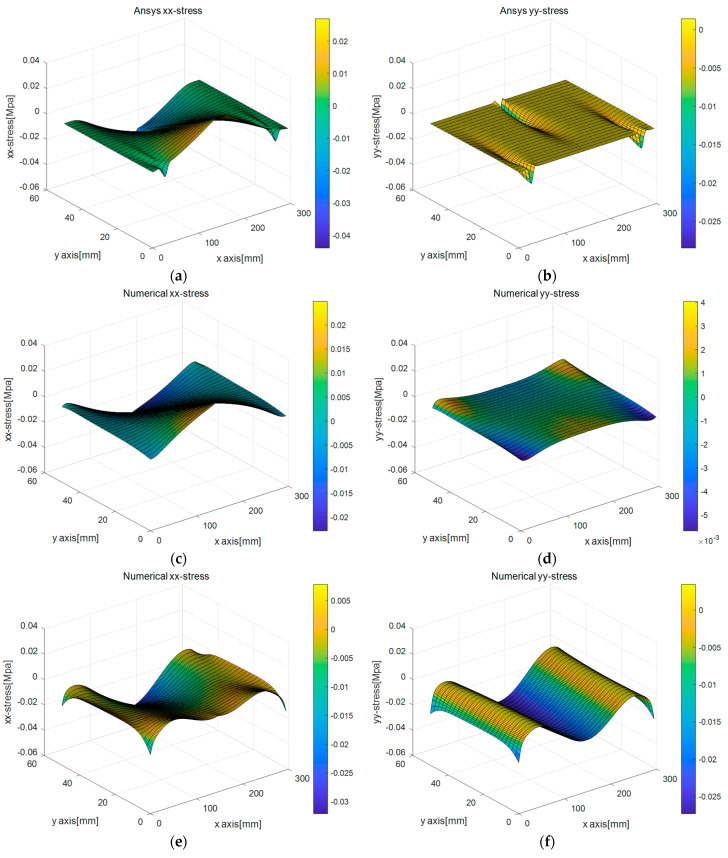

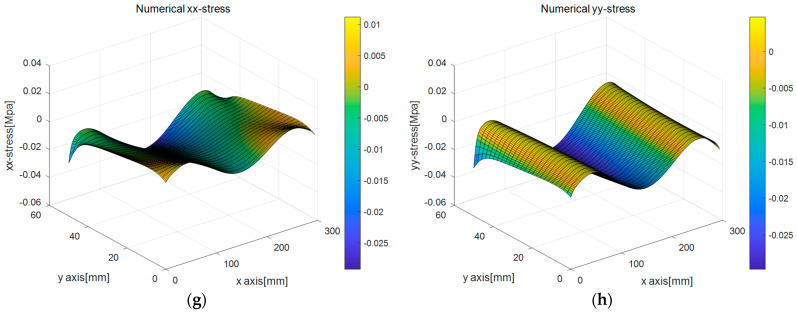
Surface plots for computed Cauchy stresses by different simulation methods and boundary capturing methods for 3-point rubber beam bending test: (**a**) $\sigma_{xx}$ (Method 1), (**b**) $\sigma_{yy}$ (Method 1), (**c**) $\sigma_{xx}$ (Method 2), (**d**) $\sigma_{yy}$ (Method 2), (**e**) $\sigma_{xx}$ (Method 3), (**f**) $\sigma_{yy}$ (Method 3), (**g**) $\sigma_{xx}$ (Method 4), (**h**) $\sigma_{yy}$ (Method 4).

**Figure 10:**
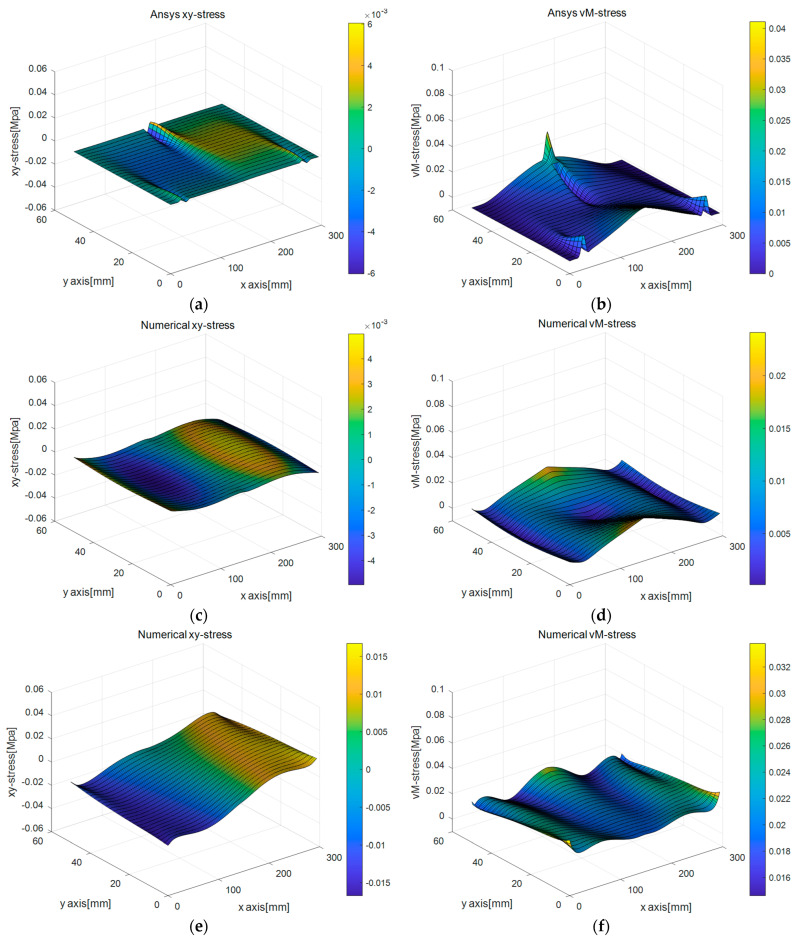

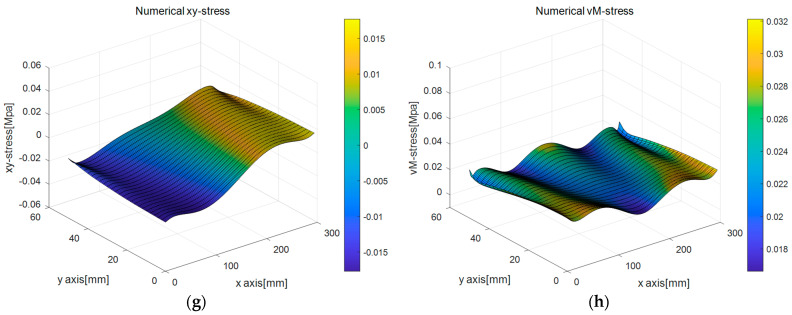
Surface plots for computed Cauchy and von Mises stress by different simulation methods and boundary capturing methods for 3-point rubber beam bending test: (**a**) $\sigma_{xy}\;\;$ (Method 1), (**b**) von Mises stress (Method 1), (**c**) $\sigma_{xy}$ (Method 2), (**d**) von Mises stress (Method 2), (**e**) $\sigma_{xy}$ (Method 3), (**f**) von Mises stress (Method 3), (**g**) $\sigma_{xy}$ (Method 4), (**h**) von Mises stress (Method 4).

It is expected that the deformation due to the bending motion dominantly occurs along the top and bottom surfaces, so the combination of the high-order regression equations and the linear strain model is used in this case. However, since it is relatively small along both side surfaces, the linear regression equations are chosen to approximate the displacement of lines 2 and 3 together with the uniform strain model. A quadratic polynomial vector and Gaussian weight function are used in the CPCM simulation, and 2 mm node spacing is applied to the CPCM model. The fifth-degree polynomials are applied for regression analysis for the top and bottom surfaces (line 1 and line 4), while the first-degree polynomials are used for both side surfaces (line 2 and line 3). Figure 8, Figure 9 and Figure 10 present the simulation results for kinematic variables, such as displacement and stress, obtained by four methods to compare the computed values. Table 1 summarizes four different combinations, and the combinations of the analysis tools and boundary capturing methods are listed. Method 1 indicates the case of full ANSYS simulation. The edge shape of the deformed specimen can be captured using the combination of DIP and polynomial regression using the deformed specimen image and the ANSYS analysis result (Method 2, 3, and 4). In Method 2, the ANSYS analysis result was only used as the data source for boundary capturing. Note that the main developments of this study are focused on Methods 3 and 4. Selection of the strain models differentiates Methods 3 and 4, i.e., Method 3 denotes the case with the linear strain model, but Method 4 involves the uniform strain model.

The surface plot for the x-directional displacement result is presented in Figure 8a–d. One reason for the erroneous behaviors found in Figure 8e–g is related to intersecting the regression functions in determining the displacements at surface line ends. This phenomenon has also been reported in the case of processing LiDAR-scanned point cloud data [36]. Although the DIP-based regression analysis has a small chance of overfitting, the slope of the estimated linear regression functions for line 2 and line 3 affects the error. The slope of the linear regression function used for line 2 and line 3 estimation in Methods 3 and 4 may differ from that used for line 2 and 3 in Method 2 since the method utilizes the ANSYS analysis result as boundary capturing source. The real experimental conditions differ from the ideally assumed conditions of ANSYS simulation. For example, in the numerical simulation, the material and the support are assumed to be linear, elastic, and frictionless at the supports. Still, real situations inevitably provoke material nonlinearity, geometric nonlinearity, and slipperiness to some extent [35]. Figure 8e,g show how the regression function for essential boundary assignment negatively affects the kinematic variable computation result; note that the regression function is determined from the intersection points, which are *x* and *y* coordinates of the corner points. Figure 8b,d,f,h represent surface plots for the computed *y*-directional displacement. It is seen that the displacements computed by the developed technique show better resolution than those obtained by DIP using LiDAR point cloud data by Seo et al. [36]. This implies that the edge points extracted from the digital image much better reflect the actual deformed shape of the loaded beam than those obtained from the point cloud data processing.

Figure 9a–h shows the surface plot for the Cauchy stress tensor ($\sigma_{xx}$, $\sigma_{yy}$) obtained by the aforementioned four different methods. As seen in Figure 9e–h, Methods 3 and 4 yield relatively poor resolution compared to Methods 1 and 2 because the resolution of capturing the essential boundary via digital image or point cloud data combined with the regression process is moderately lower than in the cases employing pure numerical simulation results. However, note that the resolution of boundary capturing is around the submillimeter level, which is practically acceptable for real-scale structural analysis. In addition, using a uniform strain model does not make a remarkable difference in the simulation accuracy. Figure 10a–h gives surface plots for shear stress and von Mises stresses, and it is noticed that they show quite similar trends to those shown in the Cauchy stress results of Figure 9a–h.

Convergence behavior as an error occurrence indicator needs to be investigated since many parts of the developed methods consist of numerical schemes. However, the DIP is difficult to evaluate in terms of the convergence concept. The conventional posteriori error analysis process, often used in computational mechanics, is adopted. The general convergence behavior of the CPCM was well investigated by Lee et al. [32] and Yoon and Song [37]. Here, the convergence rates of the CPCM combined with boundary capturing are examined. It is noted that boundary capturing provokes dominant error behavior because it generates much bigger errors than the CPCM calculation. Captured digital images should adequately reflect the actual deformed boundary of the loaded specimen. The Canny edge detection algorithm extracts sufficient edge points to involve less error and avoid overfitting in the regression process compared to the case of point cloud data processing. For the error estimation, the relative error was calculated in the following manner:

(19) $$E_{L^{2}}=\frac{{\left[\int_{\Omega }(u_{CPCM}-u_{ex}{)}^{T}(u_{CPCM}-u_{ex})d\Omega \right]}^{1/2}}{{\left[\int_{\Omega }{u_{ex}}^{T}u_{ex}d\Omega \right]}^{1/2}}$$

where $u_{CPCM}$ denotes the displacement calculated by the CPCM analysis, and $u_{ex}$ refers to the reference displacement calculated from the ANSYS analysis results. Since no exact solution is available, the FE analysis results with a refined mesh were employed as a reference solution.

**Figure 11:**
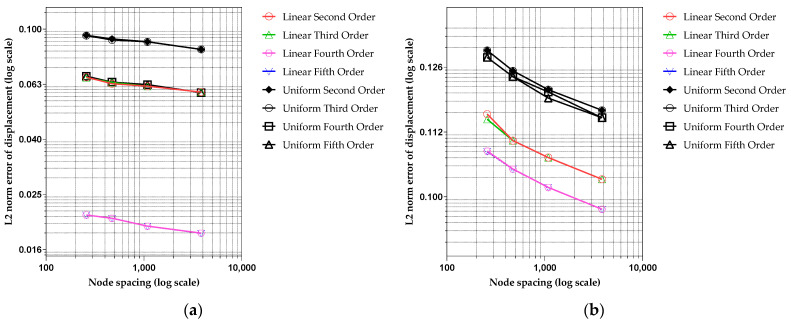
Convergence behavior of $L^{2}$ norms error (semi-log scale) for 3-point bending BVP according to various boundary capturing options such as strain model, regression function degree, and boundary capturing data source: (**a**) ANSYS-model-based boundary capturing (Methods 1 and 2); (**b**) digital-image-based boundary capturing (Method 3 and 4).

Figure 11a,b shows the convergence rates for the developed techniques regarding the relative displacement errors in a semi-log scale. Various degrees of the polynomial basis vector for CPCM and the polynomial regression function are considered in the error estimation. In the error calculation, node spacings of 8 mm (total 258 nodes), 6 mm (total 473 nodes), 4 mm (total 1089 nodes), and 2 mm (total 3848 nodes) are used. The second- to fifth-degree polynomials were applied for the regression analysis of the upper and lower surfaces (line 1 and line 4), and the linear and uniform strain models were used for boundary capturing. In Figure 11a,b, the essential boundary condition values are extracted from the ANSYS analysis results and digital images processed regression function, respectively. It is noticed that the error decreases with a moderate slope as the number of nodes increases. The absolute magnitudes of the errors are significantly small when edge points are extracted using the ANSYS analysis results because the ANSYS yields a smoother geometry of the deformed boundary than the digital image of the deformed specimen implemented in regression analysis. In addition, as seen in Figure 11a, the error size is smaller when the linear strain model is applied for the polynomial regression since it can more accurately reflect the curved edge induced by beam bending. However, the linear and uniform strain models decrease trends in the error curves as the degree of polynomial regression increases (see Figure 11b). In addition, the absolute error size significantly decreased for linear and uniform strain models when the fourth-degree polynomial regression was used. Still, there was no remarkable difference between the fourth- and fifth-degree polynomials. Although the convergence rates of the original CPCM show mathematically optimal rates according to the order of polynomial basis (see [32] and [37]), the developed method does not show a promising convergent behavior because the boundary capturing method dominates the apparent error behavior with a much bigger absolute error size than that of the original CPCM.

Regardless of the strain model, increasing the degree of polynomial regression function does not lead to overfitting but stably reduces the error. This phenomenon results from the fact that the edge points extracted from digital images are sufficient and represent a deformed beam shape well. However, it is noteworthy that only a slight difference appears between applying the second-degree polynomial with the linear strain model and the fifth-degree polynomial with the uniform strain model. Namely, when the linear strain model is used, even with a lower degree of the regression function, the error can still become smaller than the uniform strain model combined with the higher regression function. This might imply that the linear strain model can be effectively recommended in the practical boundary capturing process.

## 4. Conclusions

This study presented an image-driven hybrid structural analysis technique combining DIP for boundary capturing and the CPCM for structural analysis. The technique solves the Dirichlet-type BVP with only essential boundary conditions to analyze a deformed structure. It consists of a boundary capturing method using a DIP-based regression scheme and a strong-form-based particle method called the CPCM. Note that previously, Park et al. [35] proposed a displacement-tracking-based structural analysis where the resolution of the numerical solution depended on the density of the attached target and DIP performance because the CPCM only plays the role of interpolator using the estimated target displacements. In addition, Seo et al. [36] presented a BVP solving scheme equipped with LiDAR point cloud data and using the boundary capturing method, but the final accuracy of the scheme could not be achieved up to the mathematically optimal level because of the tricky point cloud process and the weak soundness of the LiDAR scan data.

On the other hand, to capture the deformed boundary, the new method presented in this study utilizes not the attached target or LiDAR data but the digital image. It effectively improves the boundary capturing resolution, although the basic framework of this study’s DIP technique and regression analysis are still related to those of Park et al. [35] and Seo et al. [36]. As a result, the accuracy of the BVP solving was effectively improved by the image-driven boundary capturing, which naturally led to the achievement of more accurate mechanical variables such as displacement, strain, and stress.

The effects of the regression function order and the strain model type, including the general parametric setting for the CPCM simulation, were investigated through the three-point bending test simulation of a rubber beam. For further verification, the results of the ANSYS analysis were utilized in two ways: as a reference solution for the error estimation of the convergence study and as source data for boundary capturing for comparative study in Section 3.3. The error analysis revealed that boundary capturing for the deformed specimen edge using DIP is rarely sensitive to the order of the regression function. However, in the case of boundary capturing using LiDAR-scanned point cloud data, the resolution was quite sensitive to the integrity of point cloud data [36] because the scatteredness of point cloud data for deformed edge easily makes the polynomial regression analysis quite vulnerable. On the contrary, this study showed that the pixel data derived from digital images of the deformed specimen boundary can be more consistently extracted due to the stable nature of the data source and the manipulative edge detection algorithms. Better resolutions of kinematic variables obtained by the new method were visually verified through the surface plots in Section 3.3. In addition, the convergence study demonstrated the integrity of the process as a numerical simulator. It was shown that a numerically optimal combination of the boundary capturing model and the parametric setting of the CPCM might be quantitatively determined through error analysis.

As a result, the developed methodology, which combines the image-driven regression analysis for boundary capturing with the CPCM for Dirichlet-type BVP solving, is new. Since the method enables simultaneous displacement measurement and structural analysis, it might provide new ideas for future structural health monitoring for real-scale infrastructures like concrete bridges. Furthermore, combined with deep learning techniques, the method can be dramatically improved and is expected to be applied to various engineering fields.

## Figures and Tables

**Table 1:** Combinations of analysis methods and boundary capturing techniques.

Method	Analysis Tool	Boundary Capturing Source	Regression Analysis
Method 1	Full ANSYS analysis	No boundary capturing	No regression analysis
Method 2	CPCM	ANSYS analysis result	Linear strain
Method 3	CPCM	DIP and regression analysis	Linear strain
Method 4	CPCM	DIP and regression analysis	Uniform strain

## Data Availability

Data are contained within the article.
